# Antimycobacterial Activity of Salicylanilide Benzenesulfonates

**DOI:** 10.3390/molecules17010492

**Published:** 2012-01-05

**Authors:** Martin Krátký, Jarmila Vinšová, Nabila Guisado Rodriguez, Jiřina Stolaříková

**Affiliations:** 1 Department of Inorganic and Organic Chemistry, Faculty of Pharmacy, Charles University, Heyrovského 1203, 500 05 Hradec Králové, Czech Republic; Email: martin.kratky@faf.cuni.cz (M.K.); guisadon@faf.cuni.cz (N.G.R.); 2 Laboratory for Mycobacterial Diagnostics and Tuberculosis, Regional Institute of Public Health in Ostrava, Partyzánské náměstí 7, 702 00 Ostrava, Czech Republic; Email: jirina.stolarikova@zu.cz

**Keywords:** antimycobacterial activity, benzenesulfonate, *in vitro* activity, salicylanilide ester

## Abstract

A series of eighteen novel esters of salicylanilides with benzenesulfonic acid were designed, synthesized and characterized by IR, ^1^H-NMR and ^13^C-NMR. They were evaluated *in vitro* as potential antimycobacterial agents towards *Mycobacterium tuberculosis*, *Mycobacterium avium* and two strains of *Mycobacterium kansasii*. In general, the minimum inhibitory concentrations range from 1 to 500 µmol/L. The most active compound against *M. tuberculosis* was 4-chloro-2-(4-(trifluoromethyl)phenylcarbamoyl)-phenyl benzenesulfonate, with MIC of 1 µmol/L and towards *M. kansasii* its isomer 5-chloro-2-(4-(trifluoromethyl)phenylcarbamoyl)phenyl benzenesulfonate (MIC of 2–4 µmol/L). *M. avium* was the less susceptible strain. However, generally, salicylanilide benzenesulfonates did not surpass the activity of other salicylanilide esters with carboxylic acids.

## 1. Introduction

Tuberculosis (TB) is still a global health problem. According to the WHO, in 2010 about 8.8 million people were infected by TB, including 1.1 million cases among HIV-infected patients. In the same year, 1.4 million died due to TB [1]

Recently, the increasing emergence of drug-resistant tuberculosis, especially multidrug-resistant tuberculosis (MDR-TB) and most recently extensively drug-resistant tuberculosis (XDR-TB) is alarming. A multidrug-resistant *Mycobacterium tuberculosis* strain was identified and defined as resistant to at least the two most effective anti-TB drugs—Isoniazid (INH) and rifampicin (RIF)—but it may include additional resistance to more antituberculotics. XDR-TB was defined as a resistance to any fluoroquinolone and to at least one of the three injectable drugs (capreomycin, kanamycin, or amikacin) [2,3]. In 2010, there was an estimated prevalence of 650,000 cases and about 150,000 MDR-TB deaths annually [1]. These problems and serious co-infection of TB with HIV present a serious challenge for the research to find a new type of drug or prodrug with an innovative mechanism of the action [2].

Salicylanilides (2-hydroxy-*N*-phenylbenzamides) have been revealed to demonstrate a wide range of perspective biological activities and some of them are used in human or veterinarian medicine. Salicylanilides, e.g., modulate immune response and inflammatory processes [4,5], show analgesic efficacy [6], influence on ion channels [7] and they express more molecular effects which may be useful in the development of anti-cancer drugs, e.g., references [8,9]. Salicylanilides have been studied for their significant activity against various parasites like protozoa, mycobacteria, fungi, bacteria (especially Gram-positive) or viruses [10]. Antimicrobial salicylanilides probably act by multiple mechanisms of the action [11], e.g., they disrupt membrane functions, inhibit some enzymes, block the incorporation of some nutrients [12], they act as the uncoupling agents of mitochondrial oxidative phosphorylation due to their protonophoric activity dissipating the protonmotive force [13]; from newer findings it is important that salicylanilides are able to inhibit the two-component regulatory systems which are involved in the maintenance of bacterial cell homeostasis and the expression of virulence factors [14].

Salicylanilides contain a free hydroxyl (phenolic) group generally presumed to be necessary for the activity, although its temporary masking by the esterification may be advantageous. The blockade of polar hydrophilic group can result in improved physico-chemical properties and thus better bioavailability, membrane permeability, a high activity and/or a lower toxicity and an irritant potency. In general, salicylanilide esters exhibited a similar or a sharply better antimicrobial activity than parent salicylanilides [11,15].

Salicylanilide esters as potential prodrugs have exhibited generally the very good antimycobacterial activity from 0.125 μmol/L and including MDR-TB strains [11]. Salts of benzenesulfonic acid, called also besylates, have been used in medicine as pharmaceuticals, as well as tosylates and mesylates [16]. 

In this study, we have chosen sulfonic acid instead of previously used carboxylic acids for the esterification (e.g., references [15,17,18]).

## 2. Results and Discussion

### 2.1. Chemistry

**Scheme 1:**
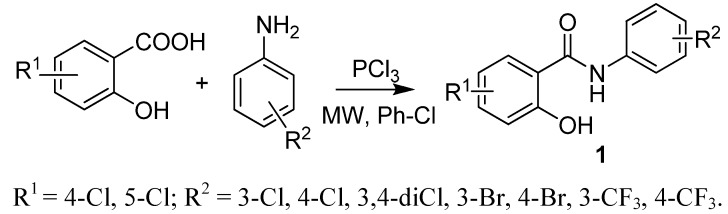
Synthesis of salicylanilides.

**Scheme 2:**
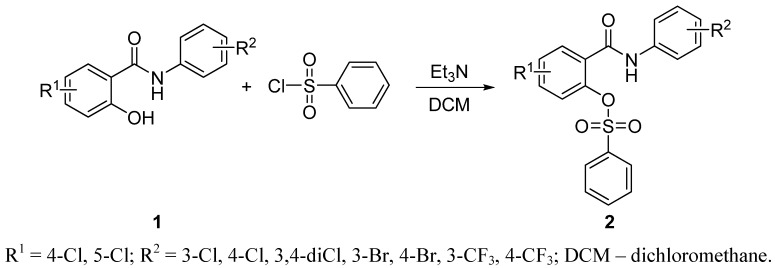
Synthesis of salicylanilide benzenesulfonates.

Salicylanilide benzenesulfonates were synthesised by two step synthesis. Firstly, salicylanilides **1** were prepared by the procedure using microwave irradiation described previously by our group, e.g., references [19] (see Scheme 1). Then they were converted to esters **2** by benzenesulfonyl chloride in the presence of triethylamine (Et_3_N; see Scheme 2); the base and solvent were chosen according to e.g., references [20]. Esterification of salicylanilides with benzenesulfonic acid *via* *N*,*N*´-dicyclohexylcarbodiimide in *N*,*N*-dimethylformamide failed.

### 2.2. *In Vitro* Antimycobacterial Evaluation

All synthesized benzenesulfonates **2** were evaluated *in vitro* towards four mycobacterial strains—*Mycobacterium tuberculosis*, *Mycobacterium avium* and two strains of *M. kansasii* after 14 and 21 days of incubation, for *M. kansasii* additionally after 7 days. The results are summarized in the Table 1.

Except for compound **2q**, for which it was not possible to determine minimal inhibition concentrations (MIC), all salicylanilide benzenesulfonates exhibited a significant *in vitro* activity against *M. tuberculosis* as the most sensitive strain at the concentration values up to 16 μmol/L. The most active derivatives were 4-chloro-2-(3,4-dichlorophenylcarbamoyl)phenyl benzenesulfonate (**2e**; 1/2 μmol/L) and 4-chloro-2-(4-(trifluoromethyl)phenylcarbamoyl)phenyl benzenesulfonate (**2q**; 1/1 μmol/L). 4-Chloro derivatives mostly showed lower MICs than their 5-chloro counterparts, in the case of **2i** and **2j**, **2k** and **2l** are both derivatives equally active. With respect to the aniline substitution pattern, the most suitable moieties are trifluoromethyl group and 3,4-dichloro substitution, whereas fluorine produced the weakest effect. With respect to the lipophilicity (calculated log*P*), there is no clear dependence, however this physico-chemical property seems to be one of the factors influencing the antimycobacterial activity. On the other side, the highest activity towards *M. tuberculosis* displayed the most lipophilic salicylanilide derivatives containing either CF_3_ group (**2o**, **2q**, **2r**; Clog*P* 5.40) or 3,4-dichloroaniline fragment (**2e**; Clog*P* 5.60) and the highest MIC exhibited the least lipophilic fluorinated esters (Clog*P* 4.64), but strongly lipophilic **2p** showed an undetectable activity.

**Table 1:** Antimycobacterial activity of salicylanilide benzenesulfonates **2**.

			MIC [μmol/L]									
	R^1^	R^2^	*M. tuberculosis* 331/88		*M. avium* 330/88		*M. kansasii* 235/80			*M. kansasii* 6509/96		
			14 d	21 d	14 d	21 d	7 d	14 d	21 d	7 d	14 d	21 d
**2a**	4-Cl	3-Cl	4	4	32	32	8	16	16	8	16	16
**2b**	5-Cl	3-Cl	16	16	500 *	500 *	16	16	16	125	500	500
**2c**	4-Cl	4-Cl	4	4	32	32	8	8	8	8	8	8
**2d**	5-Cl	4-Cl	8	8	**8**	**8**	8	16	16	8	16	16
**2e**	4-Cl	3,4-diCl	**1**	**2**	16	16	**4**	8	8	8	8	8
**2f**	5-Cl	3,4-diCl	4	8	62.5	62.5	**4**	8	8	8	8	8
**2g**	4-Cl	3-Br	8	8	16	16	16	16	16	8	8	8
**2h**	5-Cl	3-Br	16	16	250	500 *	16	62.5	62.5	32	125	125
**2i**	4-Cl	4-Br	8	8	32	32	8	8	8	8	8	16
**2j**	5-Cl	4-Br	8	8	**8**	**8**	8	16	16	**4**	8	8
**2k**	4-Cl	3-F	16	16	500 *	500 *	32	32	32	16	32	125
**2l**	5-Cl	3-F	16	16	500	500	16	32	32	16	32	32
**2m**	4-Cl	4-F	8	8	16	32	8	8	8	16	16	16
**2n**	5-Cl	4-F	16	16	32	32	16	32	32	16	16	16
**2o**	4-Cl	3-CF_3_	**2**	4	62.5	62.5	8	8	8	8	16	16
**2p**	5-Cl	3-CF_3_	125 *	125 *	125 *	125 *	125 *	125 *	125 *	125 *	125 *	125 *
**2q**	4-Cl	4-CF_3_	**1**	**1**	125	125	125 *	125 *	125 *	125 *	125 *	125 *
**2r**	5-Cl	4-CF_3_	**2**	4	250 *	250 *	**2**	**4**	**4**	**4**	**4**	**4**
**INH**			0.5	0.5	>250	>250	>250	>250	>250	2 or 4	4	8
**PAS**			62.5	62.5	32	125	125	1000	>1000	250	1000	1000
**EMB**			1	2	16	16	1	2	2	1	2	2

*M. avium* strain 330/88 resistant to INH, RIF, ofloxacin and ethambutol; INH: isoniazid; PAS: *para*-aminosalicylic acid; EMB: ethambutol. One or two lowest MIC values for each strain are bolded; *: at presented concentration the grow of strain was observed, at duplex concentration there was present precipitate and/or turbidity, therefore it was not possible to determine exact MIC value.

Salicylanilide esters of 3- or 4-bromoaniline (compounds **2g**, **2h**, **2i**, **2j**) with only a slightly lower calculated lipophilicity (Clog*P* 5.31) than trifluoromethylanilines displayed a lower average anti-mycobacterial activity than chloro derivatives (**2a**, **2b**, **2c**, **2d**; Clog*P* 5.04). Additionally, the calculated lipophilicity could not explain the differences of MIC between corresponding isomers. MIC values of the two most active derivatives **2e** and **2q** are comparable with the standards—Fist-line oral antituberculosis drugs isoniazid and ethambutol (EMB). Seventeen derivatives exhibited a significantly higher *in vitro* activity than the second-line drug *para*-aminosalicylic acid (PAS), which also shares a salicylic fragment.

Atypical mycobacteria displayed a higher rate of resistance, particularly *M. avium* with MIC value range from 8 μmol/L (**2d** and **2j**). MIC exceeded 500 μmol/L for two esters—**2b** and **2k**. The favourable substituents of the aniline ring are 4-chlorine and bromine, especially when the position 5 of the salicylic ring is occupied. In contrast to *M. tuberculosis*, a trifluoromethyl moiety brings a minimal benefit in the comparison to each other except 3-fluorine, whose derivatives showed a higher MIC of ≥500 μmol/L. Eleven benzenesulfonates certainly surpassed the activity of INH, nine of PAS and two of EMB.

Both strains of *M. kansasii* expressed an intermediate sensitivity with no distinct differences between collection and clinically isolated types. MIC for 235/80 strain lies from 2 to ≥125 μmol/L and in the range of 4–500 μmol/L for the strain 6509/96. 5-Chloro-2-(4-(trifluoromethyl)phenylcarbamoyl)-phenyl benzenesulfonate (**2r**) is the most active molecule in the tested panel with MIC of 2–4 µmol/L, *i.e.*, comparable to INH for both strains. Salicylanilide benzenesulfonates exhibited significantly higher *in vitro* activity towards *M. kansasii* 235/80 than INH, but not for 6509/96; PAS was not equally or more active than esters **2** against both strains, whereas no benzenesulfonate surpassed the MIC of EMB. Similarly to *M. avium*, the type of the salicylic ring substitution influences the activity ambiguously and the best MIC bring anilines having 4-Br or 4-Cl then 3,4-diCl substituents. The molecules with CF_3_ group showed the lowest (**2r**), moderate (**2o**) or undetected (**2p**, **2q**) MIC values. When concentrated on the position of each substituent on the aniline ring, 4-derivatives showed enhanced activity.

Salicylanilide benzenesulfonates displayed an obvious antimycobacterial activity in the range of 1–500 µmol/L with **2p** being a partial exception. In comparison with the starting salicylanilides [21], their benzenesulfonylation led to the derivatives with equal or increased MIC against atypical mycobacteria. Interestingly for *M. tuberculosis*, the modification of 5-chlorosalicylanilides (*i.e*., 4-chloro esters) resulted in a retained or an improved activity (e.g., four times for **2e**), whereas benzenesulfonates derived from 4-chlorosalicylanilides exhibited mostly similar or lower antimycobacterial activity *in vitro* than parent salicylanilides. However, when compared to other esters of carboxylic acids, they can be considered as being only moderately potent *in vitro*. Salicylanilide esters with *N*-acetyl-L-phenylalanine [15] and carbamates [22] showed markedly lower MIC than benzenesulfonates, acetates [18] demonstrated a rather superior antimycobacterial effect. Esters with various *N*-benzyloxycarbonyl α-amino acids [17] were more active against *M. tuberculosis*; the comparison of the MIC values towards *M. kansasii* appears being at similar level, however esters of some parent salicylanilides (modified in this study) as potential antimycobacterial agents were not published previously.

## 3. Experimental

### 3.1. General Methods

All used reagents and solvents were purchased from commercial sources (Sigma-Aldrich, Penta) and used without a further purification. Reactions were monitored by thin layer chromatography, plates were coated with 0.2 mm of silica gel 60 F254 (Merck) and were visualized by UV irradiation (254 nm). Melting points were determined on the Melting Point machine B-540 (Büchi) apparatus using open capillaries and they are uncorrected.

Infrared spectra (ATR) were recorded on FT-IR spectrometer Nicolet 6700 FT-IR in the range of 400–4,000 cm^−1^. The NMR spectra were measured in CDCl_3_ at ambient temperature on a Varian VNMR S500 instrument (500 MHz for ^1^H and 125 MHz for ^13^C; Varian Comp. Palo Alto, CA, USA). The chemical shifts δ are given in ppm, related to tetramethylsilane used as an internal standard. The coupling constants (*J*) are reported in Hz. Elemental analysis (C, H, N) were performed on an automatic microanalyser CHNS-O CE instrument (FISONS EA 1110, Italy). The calculated log*P* values (Clog*P*), that are the logarithm of the partition coefficient for octan-1-ol/water, were determined using the program CS ChemOffice Ultra version 11.0 (CambridgeSoft, Cambridge, MA, USA).

### 3.2. Synthesis of Salicylanilide Benzenesulfonates

An equivalent of appropriate salicylanilide (0.001 mol) was suspended with stirring in dichloromethane (DCM, 10 mL) and then triethylamine (1.5 of equivalents; 0.0015 mol) was added in one portion. The mixture was led to stir for 5 minutes to allow complete dissolution of the salicylanilide. Then benzenesulfonyl chloride (1.2 of equivalent; 0.0012 mol) was added and the mixture was stirred at the room temperature for two hours. The reaction was monitored using TLC and a toluene/methanol 9:1 mixture as eluent. After this time, the solution was evaporated till dryness, ethyl-acetate was added and the suspension was let stay at +4 °C for approximately 30 min. Then the insoluble part was removed by filtration, the filtrate was collected, partly evaporated and then added *n*-hexane to start crystallization. It was performed for 24 hours at +4 °C and the filtrates were filtered off to give esters. If necessary, they were recrystallized from boiling ethyl-acetate to afford white crystals. 

*4-Chloro-2-(3-chlorophenylcarbamoyl)phenyl benzenesulfonate* (**2a**). Yield 95%; mp 151–153 °C. IR: 3240, 1658, 1605, 1593, 1550, 1477, 1451, 1409, 1383 (SO_2_), 1323, 1202, 1177 (SO_2_), 1090, 1078, 851, 839, 781, 754, 728, 683. ^1^H-NMR: δ 8.37 (1H, s, NH), 7.85 (1H, d, *J =* 2.6 Hz, H3), 7.73 (2H, d, *J =* 7.4 Hz, H2′′, H6′′), 7.67 (1H, t, *J =* 7.5 Hz, H4′′), 7.62 (1H, t, *J =* 1.9 Hz, H2′), 7.46–7.42 (3H, m, H5, H3′′, H5′′), 7.36 (1H, dd, *J =* 8.0 Hz, *J =* 1.8 Hz, H6′), 7.27 (1H, t, *J =* 8.0 Hz, H5′), 7.24 (1H, d, *J =* 8.7 Hz, H6), 7.14 (1H, m, H4′). ^13^C-NMR: δ 160.7, 144.2, 138.4, 135.2, 134.7, 133.9, 133.8, 132.6, 131.6, 130.1, 130.0, 129.6, 128.4, 124.9, 124.8, 120.0, 117.9. Anal. Calcd. for C_19_H_13_Cl_2_NO_4_S (422.28): C, 54.04; H, 3.10; N, 3.32. Found: C, 53.82; H, 3.00; N, 3.21. Clog*P* 5.04.

*5-Chloro-2-(3-chlorophenylcarbamoyl)phenyl benzenesulfonate* (**2b**). Yield 41%; mp 164–166.5 °C. IR: 3330, 1660, 1610, 1593, 1552, 1482, 1449, 1411, 1356 (SO_2_), 1325, 1199, 1171 (SO_2_), 1095, 1070, 918, 873, 777, 751, 679, 658. ^1^H-NMR: δ 8.41 (1H, s, NH), 7.83 (1H, d, *J =* 8.4 Hz, H3), 7.75 (2H, dd, *J =* 7.5 Hz, *J =* 1.3 Hz, H2′′, H6′′), 7.67 (1H, t, *J =* 7.5 Hz, H4′′), 7.63 (1H, t, *J =* 1.8 Hz, H2′), 7.45 (2H, t, *J =* 7.8 Hz, H3′′, H5′′), 7.39–7.34 (2H, m, H4, H6′), 7.31 (1H, *J =* 2.0 Hz, H6), 7.26 (1H, t, *J =* 8.1 Hz, H5′), 7.14 (1H, m, H4′). ^13^C-NMR: δ 161.1, 146.1, 138.6, 138.2, 135.3, 134.7, 133.9, 132.7, 130.00, 129.6, 128.4, 128.2, 127.0, 124.8, 123.7, 120.0, 117.8. Anal. Calcd. for C_19_H_13_Cl_2_NO_4_S (422.28): C, 54.04; H, 3.10; N, 3.32. Found: C, 53.87; H, 3.33; N, 3.13. Clog*P* 5.04.

*4-Chloro-2-(4-chlorophenylcarbamoyl)phenyl benzenesulfonate* (**2c**). Yield 70%; mp 156–157 °C. IR: 3334, 1657, 1609, 1549, 1491, 1449, 1406, 1355 (SO_2_), 1317, 1202, 1174 (SO_2_), 1091, 828, 769, 749, 666. ^1^H-NMR: δ 8.39 (1H, s, NH), 7.85 (1H, d, *J =* 2.7 Hz, H3), 7.72 (2H, d, *J =* 7.5 Hz, H2′′, H6′′), 7.66 (1H, t, *J =* 7.5 Hz, H4′′), 7.49 (2H, d, *J =* 8.8 Hz, H2′, H6′), 7.45–7.42 (3H, m, H5, H3′′, H5′′), 7.32 (2H, d, *J =* 8.8 Hz, H3′, H5′), 7.21 (1H, d, *J =* 8.7 Hz, H6). ^13^C-NMR: δ 160.6, 144.2, 135.9, 135.2, 133.8, 132.5, 131.6, 130.1, 129.9, 129.5, 129.1, 128.4, 124.8, 121.1. Anal. Calcd. for C_19_H_13_Cl_2_NO_4_S (422.28): C, 54.04; H, 3.10; N, 3.32. Found: C, 54.25; H, 3.36; N, 3.14. Clog*P* 5.04.

*5-Chloro-2-(4-chlorophenylcarbamoyl)phenyl benzenesulfonate* (**2d**). Yield 97%; mp 152.5–155 °C. IR: 3339, 1658, 1608, 1596, 1548, 1490, 1450, 1403, 1355 (SO_2_), 1319, 1198, 1170 (SO_2_), 1086, 1069, 917, 830, 768, 750, 683, 656. ^1^H-NMR: δ 8.41 (1H, s, NH), 7.84 (1H, d, *J =* 8.5 Hz, H3), 7.73 (2H, d, *J =* 7.6 Hz, H2′′, H6′′), 7.66 (1H, t, *J =* 7.5 Hz, H4′′), 7.50 (2H, d, *J =* 8.8 Hz, H2′, H6′), 7.44 (2H, t, *J =* 7.0 Hz, H3′′, H5′′), 7.38 (1H, dd, *J =* 8.4 Hz, *J =* 2.0, H4), 7.31 (2H, d, *J =* 8.9 Hz, H3′, H5′), 7.29 (1H, *J =* 1.9 Hz, H6). ^13^C-NMR: 161.0, 146.1, 138.1, 136.1, 135.3, 133.8, 132.7, 129.7, 129.5, 129.1, 128.4, 128.3, 127.1, 123.7, 121.1. Anal. Calcd. for C_19_H_13_Cl_2_NO_4_S (422.28): C, 54.04; H, 3.10; N, 3.32. Found: C, 53.93; H, 2.88; N, 3.54. Clog*P* 5.04.

*4-Chloro-2-(3,4-dichlorophenylcarbamoyl)phenyl benzenesulfonate* (**2e**). Yield 72%; mp 158–160 °C. IR: 3333, 1661, 1608, 1596, 1542, 1475, 1450, 1401, 1353 (SO_2_), 1319, 1198, 1175 (SO_2_), 1138, 1089, 850, 827, 779, 752, 670. ^1^H-NMR: δ 8.47 (1H, s, NH), 7.84 (1H, d, *J =* 2.6 Hz, H3), 7.75 (1H, d, *J =* 2.4 Hz, H2′), 7.74 (2H, d, *J =* 8.1 Hz, H2′′, H6′′), 7.69 (1H, t, *J =* 7.5 Hz, H4′′), 7.49–7.43 (3H, m, H5, H3′′, H5′′), 7.41–7.33 (2H, m, H5′, H6′), 7.18 (1H, d, *J =* 8.7 Hz, H6). ^13^C-NMR: δ 160.8, 144.2, 136.8, 135.3, 133.9, 133.8, 132.8, 132.7, 131.6, 130.6, 129.9, 129.6, 128.4, 128.1, 124.8, 121.6, 119.1. Anal. Calcd. for C_19_H_12_Cl_3_NO_4_S (456.73): C, 49.96; H, 2.65; N, 3.07. Found: C, 50.24; H, 2.91; N, 2.99. Clog*P* 5.60.

*5-Chloro-2-(3,4-dichlorophenylcarbamoyl)phenyl benzenesulfonate* (**2f**). Yield 63%; mp 164–164.5 °C. IR: 3316, 3107, 1664, 1587, 1533, 1477, 1449, 1399, 1353 (SO_2_), 1312, 1200, 1171 (SO_2_), 1127, 1094, 1067, 908, 815, 769, 757, 684, 662. ^1^H-NMR: δ 8.42 (1H, s, NH), 7.84 (1H, d, *J =* 8.4 Hz, H3), 7.76–7.73 (3H, m, H2′, H2′′, H6′′), 7.70 (1H, t, *J =* 8.1 Hz, H4′′), 7.48 (2H, t, *J =* 7.7 Hz, H3′′, H5′′), 7.41–7.36 (2H, m, H5′, H6′), 7.34 (1H, dd, *J =* 8.5 Hz, *J =* 2.0, H4), 7.26 (1H, *J =* 2.3 Hz, H6). ^13^C-NMR: δ 161.2, 146.1, 138.5, 136.9, 135.4, 133.9, 132.9, 132.8, 130.6, 129.7, 128.4, 128.3, 128.0, 126.8, 123.7, 121.6, 119.1. Anal. Calcd. for C_19_H_12_Cl_3_NO_4_S (456.73): C, 49.96; H, 2.65; N, 3.07. Found: C, 49.83; H, 2.87; N, 3.24. Clog*P* 5.60.

*2-(3-Bromophenylcarbamoyl)-4-chlorophenyl benzenesulfonate* (**2g**). Yield 88%; mp 141.5–143.5 °C. IR: 3239, 3078, 1659, 1605, 1587, 1548, 1477, 1450, 1406, 1382 (SO_2_), 1322, 1201, 1177 (SO_2_), 1089, 1071, 871, 848, 839, 779, 754, 728, 682. ^1^H-NMR: δ 8.34 (1H, s, NH), 7.85 (1H, d, *J =* 2.8 Hz, H3), 7.76–7.72 (3H, m, H2′, H2′′, H6′′), 7.67 (1H, t, *J =* 7.5 Hz, H4′′), 7.47‑7.40 (4H, m, H5, H6′, H3′′, H5′′), 7.31–7.25 (2H, m, H4′, H5′), 7.24 (1H, d, *J =* 8.9 Hz, H6). ^13^C-NMR: δ 160.7, 144.2, 138.6, 135.2, 133.9, 133.8, 132.6, 131.6, 130.3, 130.0, 129.6, 128.4, 127.9, 124.9, 122.8, 122.7, 118.3. Anal. Calcd. for C_19_H_13_BrClNO_4_S (466.73): C, 48.89; H, 2.81; N, 3.00. Found: C, 48.99; H, 2.58; N, 2.76. Clog*P* 5.31.

*2-(3-Bromophenylcarbamoyl)-5-chlorophenyl benzenesulfonate* (**2h**). Yield 79%; mp 169.5–172 °C. IR: 3330, 1660, 1609, 1588, 1550, 1478, 1449, 1407, 1355 (SO_2_), 1324, 1198, 1170 (SO_2_), 1093, 1071, 916, 862, 776, 767, 750, 679, 662. ^1^H-NMR: δ 8.38 (1H, bs, NH), 7.84 (1H, d, *J =* 9.0 Hz, H3), 7.77–7.73 (3H, m, H2′, H2′′, H6′′), 7.67 (1H, t, *J =* 8.4 Hz, H4′′), 7.48‑7.23 (7H, m, H4, H6, H4′, H5′, H6′, H3′′, H5′′). ^13^C-NMR: δ 161.1, 146.1, 138.7, 138.2, 135.3, 133.9, 132.7, 130.3, 129.6, 128.4, 128.3, 127.7, 127.0, 123.8, 122.8, 122.6, 118.3. Anal. Calcd. for C_19_H_13_BrClNO_4_S (466.73): C, 48.89; H, 2.81; N, 3.00. Found: C, 49.12; H, 2.71; N, 3.15. Clog*P* 5.31.

*2-(4-Bromophenylcarbamoyl)-4-chlorophenyl benzenesulfonate* (**2i**). Yield 50%; mp 160–160.5 °C. IR: 3341, 1660, 1607, 1591, 1547, 1487, 1449, 1400, 1354 (SO_2_), 1317, 1197, 1169 (SO_2_), 1093, 1073, 916, 828, 767, 750, 683, 654. ^1^H-NMR: δ 8.40 (1H, s, NH), 7.85 (1H, d, *J =* 2.7 Hz, H3), 7.72 (2H, d, *J =* 7.5 Hz, H2′′, H6′′), 7.66 (1H, t, *J =* 7.5 Hz, H4′′), 7.48–7.41 (6H, m, H2′, H3′, H5′, H6′, H3′′, H5′′) 7.37 (1h, dd, *J =* 6.6 Hz, *J =* 1.9 Hz, H4), 7.22 (1H, d, *J =* 8.8 Hz, H6). ^13^C-NMR: δ 160.7, 144.2, 136.6, 135.1, 133.9, 133.8, 132.6, 131.6, 130.2, 129.6, 128.4, 124.8, 123.6, 121.4, 117.4. Anal. Calcd. for C_19_H_13_BrClNO_4_S (466.73): C, 48.89; H, 2.81; N, 3.00. Found: C, 48.63; H, 2.92; N, 3.27. Clog*P* 5.31.

*2-(4-Bromophenylcarbamoyl)-5-chlorophenyl benzenesulfonate* (**2j**). Yield 96%; mp 155.5–157.5 °C. IR: 3341, 1660, 1607, 1591, 1547, 1487, 1449, 1400, 1354 (SO_2_), 1317, 1198, 1169 (SO_2_), 1093, 1070, 916, 828, 767, 750, 683, 654. ^1^H-NMR: δ 8.38 (1H, s, NH), 7.84 (1H, d, *J =* 8.4 Hz, H3), 7.73 (2H, dd, *J =* 8.5 Hz, *J =* 1.2 Hz, H2′′, H6′′), 7.67 (1H, tt, *J =* 7.2 Hz, *J =* 1.2 Hz, H4′′), 7.47‑7.41 (6H, m, H2′, H3′, H5′, H6′, H3′′, H5′′), 7.38 (1H, dd, *J =* 8.4 Hz, *J =* 2.0, H4), 7.30 (1H, *J =* 2.0 Hz, H6). ^13^C-NMR: δ 161.0, 146.1, 138.1, 136.6, 135.3, 133.8, 132.7, 132.0, 129.6, 128.4, 128.2, 127.1, 123.7, 121.4, 117.4. Anal. Calcd. for C_19_H_13_BrClNO_4_S (466.73): C, 48.89; H, 2.81; N, 3.00. Found: C, 48.76; H, 2.60; N, 2.78. Clog*P* 5.31.

*4-Chloro-2-(3-fluorophenylcarbamoyl)phenyl benzenesulfonate* (**2k**). Yield 65%; mp 136–138.5 °C. IR: 3330, 1660, 1613, 1555, 1480, 1447, 1356 (SO_2_), 1293, 1266, 1201, 1177 (SO_2_), 1090, 1075, 844, 782, 746, 681. ^1^H-NMR: δ 8.42 (1H, s, NH), 7.85 (1H, d, *J =* 2.7 Hz, H3), 7.72 (2H, dd, *J =* 8.5 Hz, *J =* 1.1 Hz, H2′′, H6′′), 7.66 (1H, dt, *J =* 7.5 Hz, *J =* 1.1 Hz, H4′′), 7.51 (1H, dt, *J =* 8.1 Hz, *J =* 2.2 Hz, H5), 7.47‑7.41 (3H, m, H2′, H3′′, H5′′), 7.30 (1H, dd, *J =* 8.1 Hz, *J =* 1.7 Hz, H6′), 7.25 (1H, t, *J =* 7.8 Hz, H5′), 7.16 (1H, d, *J =* 8.1 Hz, H6), 6.97 (1H, td, *J =* 8.3 Hz, *J =* 2.7 Hz, H4′). ^13^C-NMR: δ 163.9 and 161.9 (*J =* 245.3 Hz), 160.7, 144.2, 138.9 and 138.8 (*J =* 11.1 Hz), 135.2, 133.8, 132.6, 131.6, 130.2 and 130.1 (*J =* 9.4 Hz), 130.1, 129.5, 128.4, 128.2, 124.8, 115.2 and 115.1 (*J =* 3.0 Hz), 111.7 and 111.5 (*J =* 21.5 Hz), 107.5 and 107.3 (*J =* 26.8 Hz). Anal. Calcd. for C_19_H_13_ClFNO_4_S (405.83): C, 56.23; H, 3.23; N, 3.45. Found: C, 56.51; H, 3.01; N, 3.68. Clog*P* 4.64.

*5-Chloro-2-(3-fluorophenylcarbamoyl)phenyl benzenesulfonate* (**2l**). Yield 95%; mp 134.5–137 °C. IR: 3332, 1661, 1613, 1599, 1553, 1491, 1448, 1426, 1356 (SO_2_), 1328, 1199, 1174 (SO_2_), 1149, 1093, 1066, 914, 862, 776, 748, 681, 665. ^1^H-NMR: δ 8.45 (1H, s, NH), 7.82 (1H, d, *J =* 8.4 Hz, H3), 7.74 (2H, dd, *J =* 8.5 Hz, *J =* 1.7 Hz, H2′′, H6′′), 7.67 (1H, dt, *J =* 8.6 Hz, *J =* 1.7 Hz, H4′′), 7.49–7.41 (3H, m, H2′, H3′′, H5′′), 7.37 (1H, dd, *J =* 8.5 Hz, *J =* 2.0 Hz, H4), 7.31–7.26 (2H, m, H6, H6′), 7.25 (1H, t, *J =* 8.0 Hz, H5′), 6.86 (1H, td, *J =* 8.2 Hz, *J =* 2.6 Hz, H4′). ^13^C-NMR: δ 163.9 and 161.9 (*J =* 245.0 Hz), 161.1, 146.1, 139.0 and 138.9 (*J =* 10.8 Hz), 138.2, 135.2, 133.8, 132.6, 130.2 and 130.1 (*J =* 9.2 Hz), 129.5, 128.4, 128.2, 127.1, 123.7, 115.1 and 115.1 (*J =* 2.9 Hz), 111.5 and 111.4 (*J =* 21.4 Hz), 107.5 and 107.3 (*J =* 26.6 Hz). Anal. Calcd. for C_19_H_13_ClFNO_4_S (405.83): C, 56.23; H, 3.23; N, 3.45. Found: C, 55.97; H, 3.45; N, 3.64. Clog*P* 4.64.

*4-Chloro-2-(4-fluorophenylcarbamoyl)phenyl benzenesulfonate* (**2m**). Yield 82%; mp 111–113.5 °C. IR: 3255, 3079, 1653, 1619, 1562, 1508, 1475, 1451, 1411, 1382 (SO_2_), 1321, 1213, 1202, 1177 (SO_2_), 1091, 849, 834, 793, 770, 729, 710, 683. ^1^H-NMR: δ 8.37 (1H, s, NH), 7.85 (1H, d, *J =* 2.6 Hz, H3), 7.72 (2H, dd, *J =* 8.5 Hz, *J =* 1.1 Hz, H2′′, H6′′), 7.65 (1H, t, *J =* 7.5 Hz, H4′′), 7.52–7.49 (2H, m, H2′, H6′), 7.45–7.41 (3H, m, H5, H3′′, H5′′), 7.19 (1H, d, *J =* 8.8 Hz, H6), 7.06–7.02 (2H, m, H3′, H5′). ^13^C-NMR: δ 160.6, 160.5 and 158.6 (*J =* 242.8 Hz), 144.2, 135.1, 134.0, 133.8, 133.4 and 133.4 (*J =* 2.8 Hz), 132.4, 131.5, 130.3, 129.5, 128.4, 124.7, 121.7 and 121.7 (*J =* 7.9 Hz), 115.8 and 115.6 (*J =* 22.5 Hz). Anal. Calcd. for C_19_H_13_ClFNO_4_S (405.83): C, 56.23; H, 3.23; N, 3.45. Found: C, 56.01; H, 3.04; N, 3.69. Clog*P* 4.64.

*5-Chloro-2-(4-fluorophenylcarbamoyl)phenyl benzenesulfonate* (**2n**). Yield 91%; mp 122.5–124 °C. IR: 3331, 1658, 1616, 1597, 1556, 1506, 1479, 1449, 1410, 1354 (SO_2_), 1328, 1201, 1177 (SO_2_), 1095, 1068, 916, 887, 836, 783, 771, 755, 683. ^1^H-NMR: δ 8.38 (1H, s, NH), 7.84 (1H, d, *J =* 9.0 Hz, H3), 7.74 (2H, d, *J =* 7.4 Hz, H2′′, H6′′), 7.66 (1H, t, *J =* 7.5 Hz, H4′′), 7.52–7.49 (2H, m, H2′, H6′), 7.44 (2H, t, *J =* 7.9 Hz, H3′′, H5′′), 7.37 (1H, dd, *J =* 8.4 Hz, *J =* 1.9, H4), 7.27 (1H, *J =* 1.9 Hz, H6), 7.06‑7.02 (2H, m, H3′, H5′). ^13^C-NMR: δ 161.0, 159.4 and 157.5 (*J =* 240.5 Hz), 146.1, 138.0, 135.2, 134.8, 133.5 and 133.5 (*J =* 2.8 Hz), 132.6, 129.5, 128.4, 128.2, 127.3, 123.6, 121.7 and 121.6 (*J =* 7.8 Hz), 115.8 and 115.6 (*J =* 22.6 Hz). Anal. Calcd. for C_19_H_13_ClFNO_4_S (405.83): C, 56.23; H, 3.23; N, 3.45. Found: C, 56.47; H, 3.40; N, 3.32. Clog*P* 4.64.

*4-Chloro-2-(3-(trifluoromethyl)phenylcarbamoyl)phenyl benzenesulfonate* (**2o**). Yield 51%; mp 118.5–119.5 °C. IR: 3335, 1659, 1615, 1561, 1493, 1471, 1450, 1397, 1353 (SO_2_), 1336, 1314, 1200, 1168 (SO_2_), 1111, 1092, 1072, 890, 849, 800, 782, 747, 690, 657. ^1^H-NMR: δ 8.46 (1H, s, NH), 7.89 (1H, d, *J =* 2.7 Hz, H3), 7.84 (1H, s, H2′), 7.75 (2H, d, *J =* 8.7 Hz, *J =* 1.3 Hz, H2′′, H6′′), 7.71-7.63 (2H, m, H6′, H4′′), 7.52–7.41 (5H, m, H5, H4′, H5′, H3′′, H5′′), 7.26 (1H, d, *J =* 8.7 Hz, H6). ^13^C-NMR: δ 160.9, 144.3, 137.9, 135.3, 134.0, 133.9, 132.7, 131.6, 131.3, 129.9, 129.7, 129.6, 128.4, 124.4, 122.9, 121.9, 121.4 (q, *J =* 3.8 Hz), 116.7 (q, *J =* 3.9 Hz). Anal. Calcd. for C_20_H_13_ClF_3_NO_4_S (455.83): C, 52.70; H, 2.87; N, 3.07. Found: C, 53.02; H, 3.05; N, 2.87. Clog*P* 5.40.

*5-Chloro-2-(3-(trifluoromethyl)phenylcarbamoyl)phenyl benzenesulfonate* (**2p**). Yield 42%; mp 175–176 °C. IR: 3330, 1661, 1615, 1597, 1573, 1562, 1493, 1450, 1397, 1354 (SO_2_), 1337, 1316, 1198, 1170 (SO_2_), 1116, 1096, 1072, 920, 873, 796, 769, 751, 698, 658. ^1^H-NMR: δ 8.45 (1H, s, NH), 7.89 (1H, d, *J =* 8.5 Hz, H3), 7.85 (1H, s, H2′), 7.76 (2H, d, *J =* 8.5 Hz, *J =* 1.1 Hz, H2′′, H6′′), 7.67 (1H, dt, *J =* 7.5 Hz, *J =* 1.2 Hz, H4′′), 7.51–7.39 (6H, m, H5, H4′, H5′, H6′, H3′′, H5′′), 7.33 (1H, d, *J =* 2.0 Hz, H6). ^13^C-NMR: δ 161.3, 146.1, 138.4, 138.0, 135.3, 134.0, 132.8, 131.7, 131.3, 129.8, 129.6, 128.4, 126.8, 125.6, 123.8, 122.9, 121.4 (q, *J =* 4.0 Hz), 116.7 (q, *J =* 3.7 Hz). Anal. Calcd. for C_20_H_13_ClF_3_NO_4_S (455.83): C, 52.70; H, 2.87; N, 3.07. Found: C, 53.02; H, 3.05; N, 2.87. Clog*P* 5.40.

*4-Chloro-2-(4-(trifluoromethyl)phenylcarbamoyl)phenyl benzenesulfonate* (**2q**). Yield 81%; mp 175.5–177 °C. IR: 3335, 1663, 1609, 1552, 1472, 1450, 1414, 1355 (SO_2_), 1321, 1200, 1171 (SO_2_), 1100, 1090, 1066, 839, 773, 747, 669. ^1^H-NMR: δ 8.55 (1H, s, NH), 7.88 (1H, d, *J =* 2.7 Hz, H3), 7.73 (2H, dd, *J =* 8.5 Hz, *J =* 1.3 Hz, H2′′, H6′′), 7.69‑7.61 (5H, m, H2′, H3′, H5′, H6′, H4′′), 7.48–7.42 (3H, m, H5, H3′′, H5′′), 7.21 (1H, d, *J =* 8.7 Hz, H6). ^13^C-NMR: δ 160.9, 144.3, 140.4, 135.3, 134.0, 133.9, 132.7, 131.7, 129.9, 129.6, 128.4, 126.5 (q, *J =* 32.8 Hz), 126.4 (q, *J =* 3.8 Hz), 124.8, 124.0 (q, *J =* 271.6 Hz), 119.6. Anal. Calcd. for C_20_H_13_ClF_3_NO_4_S (455.83): C, 52.70; H, 2.87; N, 3.07. Found: C, 52.54; H, 2.99; N, 3.23. Clog*P* 5.40.

*5-Chloro-2-(4-(trifluoromethyl)phenylcarbamoyl)phenyl benzenesulfonate* (**2r**). Yield 96%; mp 164–166.5 °C. IR: 3336, 1665, 1607, 1552, 1478, 1449, 1412, 1354 (SO_2_), 1322, 1198, 1168 (SO_2_), 1106, 1065, 917, 846, 769, 751, 683, 659. ^1^H-NMR: δ 8.60 (1H, s, NH), 7.84 (1H, d, *J =* 8.7 Hz, H3), 7.74 (2H, dd, *J =* 8.5 Hz, *J =* 1.1 Hz, H2′′, H6′′), 7.69–7.59 (5H, m, H2′, H3′, H5′, H6′, H4′′), 7.44 (2H, t, *J =* 8.0 Hz, H3′′, H5′′), 7.39 (1H, dd, *J =* 8.4 Hz, *J =* 1.9, H4), 7.27 (1H, *J =* 1.9 Hz, H6). ^13^C- NMR: δ 161.4, 146.1, 140.5, 138.4, 135.4, 133.8, 132.7, 129.6, 128.4, 128.3, 127.0, 126.5 (q, *J =* 32.8 Hz), 126.3 (q, *J =* 3.9 Hz), 124.0 (q, *J =* 271.6 Hz), 123.7, 119.6. Anal. Calcd. for C_20_H_13_ClF_3_NO_4_S (455.83): C, 52.70; H, 2.87; N, 3.07. Found: C, 52.88; H, 3.11; N, 3.25. Clog*P* 5.40.

### 3.3. Antimycobacterial Susceptibility Testing

All the prepared compounds were tested for their *in vitro* antimycobacterial activity in the Laboratory for Mycobacterial Diagnostics and Tuberculosis, Ostrava, against *M. tuberculosis* 331/88 (H37Rv) (dilution of the strain was 10^−3^) and moreover for some non-tuberculous INH-resistant strains *M. avium* (330/88, resistant to INH, rifampicin, ofloxacin, and ethambutol; dilution 10^−5^) and *M. kansasii* (235/80, dilution 10^−4^). One strain was clinically isolated *M. kansasii* 6509/96 (dilution 10^−5^); other strains were obtained from the Czech National Collection of Type Cultures (CNCTC). The micromethod for the determination of the minimum inhibitory concentration (MIC) was used. Antimycobacterial activities were determined in the Šula semisynthetic medium [23] (SEVAC, the Czech Republic). The tested compounds were added to the medium as solutions in dimethyl sulfoxide (DMSO). The following concentrations were used: 1000, 500, 250, 125, 62, 32, 16, 8, 4, 2, 1, 0.5, 0.25, and 0.125 µmol/L. The MICs were determined after incubation at 37 °C for 14 and 21 days, for *M. kansasii* additionally for 7 days. MIC (µmol/L) was the lowest concentration at which the complete inhibition of mycobacterial growth occurred. As the reference compound were chosen the first-line antituberculosis drugs isoniazid (INH), ethambutol (EMB) and the second-line drug *para*-aminosalicylic acid (PAS) sharing a partial structure similarity with presented derivatives.

## 4. Conclusions

In sum, we have designed and synthesized eighteen new salicylanilide benzenesulfonates *via* treatment of the triethylammonium salts of salicylanilides with benzenesulfonyl chloride. They were characterised and evaluated as potential antimycobacterial agents. With one exception, all derivatives affected the growth of *M. tuberculosis* at 1–16 µmol/L as well as atypical strains, albeit at higher concentrations. Salicylanilide benzenesulfonates fulfilled the expectation about their antimycobacterial activity, although, in general, at higher concentrations than salicylanilide esters with carboxylic acids.
